# Optimization on Reducing Slag Entrapment in 150 × 1270 mm Slab Continuous Casting Mold

**DOI:** 10.3390/ma12111774

**Published:** 2019-05-31

**Authors:** Yang Wang, Shufeng Yang, Feng Wang, Jingshe Li

**Affiliations:** 1 School of Metallurgical and Ecological Engineering, University of Science and Technology Beijing, Beijing 100083, China; ustb_wang@163.com (Y.W.); lijingshe@ustb.edu.cn (J.L.); 2 Institute of Engineering Technology, University of Science and Technology Beijing, Beijing 100083, China; wangfeng02@hbisco.com

**Keywords:** continuous casting, mold, slag entrapment, numerical modelling, process simulations, fluid dynamics

## Abstract

To reduce slag entrapment in 150 × 1270 mm slab continuous casting molds at the Tang Steel Company, the effect of submerged entrance nozzle (SEN) depth and casting speed on the phenomenon was studied by computational fluid dynamics simulations. Then, the slag entrapment behavior in continuous casting molds, utilizing Large Eddy Simulation (LES) by coupling the volume of fluid (VOF) method, was also used. Finally, the effect of several common oils usually used to simulate slag in water modelling on slag entrapment was discussed and the water modelling results were used to validate the numerical simulation findings. The results showed that the optimum scheme is a submerged depth of SEN 90 mm and a casting speed of 1.6 m/min. Under optimal conditions, the maximum surface velocity is smallest (0.335 m/s) and the maximum slag entrapment ratio (0.44%) appears in the position of 0.1 m below the meniscus after 15 s. The water modelling results were in good agreement with the numerical simulation results.

## 1. Introduction

The core concept of high efficiency continuous casting is to produce high quality defect-free steel at high casting speeds. At high casting speeds, slag entrapment in molds seriously deteriorate the quality of steel products, becoming one of the main obstacles affecting the production of high value-added steel products [1].

**Figure 1:**
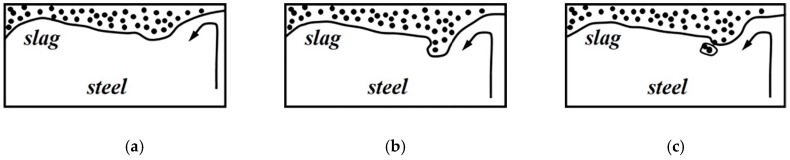
Slag entrapment by shear–layer instability: (**a**) flow upward after stream striking narrow face, (**b**) shearing effect on slag layer, (**c**) formation of slag droplet.

Numerous researchers [2,3,4,5,6,7] have studied slag entrapment in the mold region of the continuous casting process using water modelling. Thomas et al. [8] have summarized that mold slag entrainment could cause both surface and internal defects in final products if the entrained droplets become trapped in the solidifying metal, which makes it a significant problem in the production of clean steel. Further analysis indicates three kinds of mold slag entrapment mechanisms: Vortexing, shear force, and turbulence at the meniscus. Iguchi et al. [9] focused on the shear flow instability between molten steel flow and mold powder as one of causes for mold powder entrapment, and investigated the effect of kinematic viscosity of mold powder on the onset of entrapment. However, they did not explain why they chose salt water, which has a different density ratio to water. However, the role of the steel–slag interface has only seen minimal investigation. When a liquid–liquid interface is exposed to a shear force from a flow initiated by a rotating roller, the meniscus is deformed. The flow acts tangentially on the interface leading to the formation of a finger-like protrusion, as shown in Figure 1. The interfacial tension acts against the tendency for droplet entrainment. In order to describe the flow-induced entrainment of lighter phase droplets, dimensionless relations are derived. The critical flow velocity (u) for entrainment is an important parameter for slag entrapment in continuous casting molds.

Thomas et al. [8] mentioned that the interface between two density-stratified fluids with relative motion will become unstable with a sufficiently large difference in velocity. Most studies of slag entrainment have identified this phenomenon, known as Kelvin–Helmholtz instability (KHI), as a cause of mold slag entrainment, as shown in Figure 1. This shear instability mechanism is most likely to occur halfway between the narrow face and the submerged entrance nozzle (SEN), where the horizontal surface velocity is largest. Helmholtz first explored the theoretical condition for the instability [10], and an alternative prediction [11] of the Kelvin–Helmholtz instability for finite layer thickness, inviscid fluids and zero interfacial tension gives a critical velocity at the same time. Jari et al. [12] obtained a modified Weber number through experimentation. The modified Weber number could be used as a criterion for droplet formation from the oil layer. However, it does not include oil viscosity, which makes it insufficient criterion for slag entrainment.

Another important dimensionless quantity in slag entrapment is the capillary number, which is defined by the ratio of deforming stress exerted by a continuous liquid and the counteracting Laplace pressure [13,14]. However, there are no comprehensive similarity criteria with regards to what kind of oil should be used in a water model to properly represent the slag entrapment occurring in a continuous casting mold. The influence of the density ratio on critical velocity was noted in a previous study where the values of critical velocity obtained were assumed without justification to be smaller than in a real steel-slag system [3,15].

In actual production, SEN depth and casting speed in continuous casting process are two direct impact factors on the shear-layer instability and further affect the occurrence of slag entrapment. To reduce the slag entrapment in a 150 × 1270 mm slab continuous casting mold at the Tang Steel Company, in this paper, the optimum submerged depth of SEN and casting speed were studied using numerical simulations. Then, slag entrapment in the mold using computational fluid dynamics simulations, utilizing the LES (Large Eddy Simulation) coupling volume of fluid (VOF) method, was also used. Finally, the effect of several common oils usually used to simulate slag in water modelling on slag entrapment was discussed and the water modelling results were used to validate the numerical simulation findings.

## 2. Experimental Section

### 2.1. Experimental Setup

**Figure 2:**
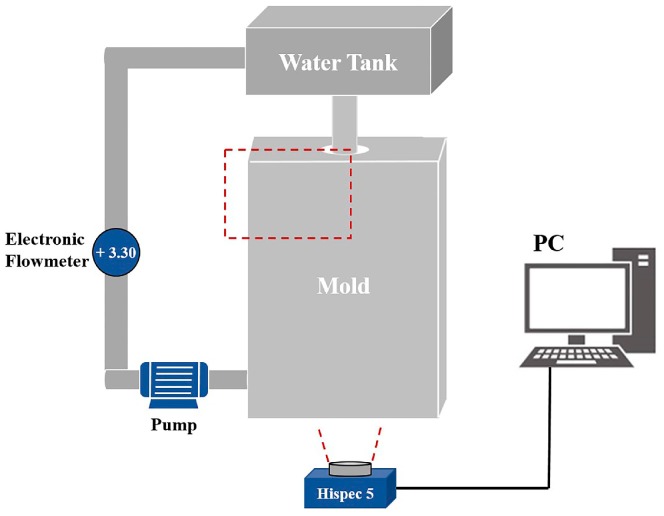
Schematic drawing of the experimental apparatus.

Figure 2 shows a schematic of the full-scale water modelling experimental apparatus, consisting of mold with a submerged nozzle, water tank, electronic flowmeter, pump and high-speed camera.

The bottom of the mold was connected to the water tank on the top through water pipes. After flowing out of the outlet, water was pumped back to the top tank. A high-speed camera in front of the zone, shown as a red dotted line, was used to record fluctuations of the water–oil interface and the entrapment of oil droplets. For each experimental work with the water–oil system, both liquids were renewed.

**Figure 3:**
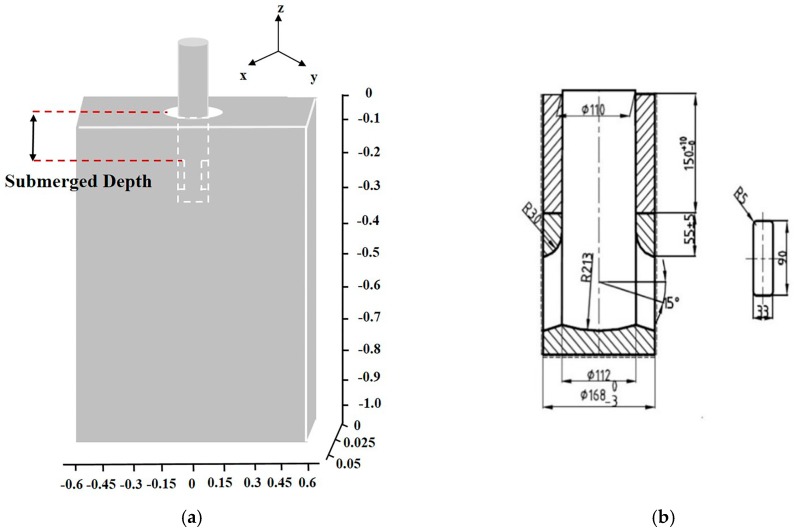
Schematic of mold water model: (**a**) mold size and (**b**) submerged entrance nozzle size.

The mold geometry is shown in Figure 3a. A straight-mold steel caster of a 150-mm-thick and 1270-mm-wide strand is modeled with a water model that is 1100 mm in length. The example submerged entrance nozzle configuration is also shown in Figure 3b.

### 2.2. Similarity Criterion and Dimensional Analysis

The critical flow velocity U* for slag entrapment is a function of the material properties of both phases:

(1) $$U^{*}=f\left\{\rho_{1},\rho_{2},\eta_{1},\eta_{2},\delta ,g\right\}$$

where *η*_1_ and *η*_2_ are the viscosities (Pa·s), *ρ*_1_ and *ρ*_2_ are the densities of the lighter and heavier phases in contact (kg/m^3^). g is the acceleration of gravity (m/s^2^) and δ is the interface tension force (N/m).

According to the Buckingham *π* theorem, it is possible to achieve four *π* groups:

(2) $$\pi_{1}=f\{\pi_{2},\pi_{3},\pi_{4}\}$$

Selecting *η*_2_, *ρ*_2_, and *u* as basic quantities, the following could be obtained:

(3) $$\begin{array}{l} \left[\eta_{2}\right]=\left[M^{1},L^{-1},T^{-1}\right]\\ \left[u\right]=\left[M^{0},L^{1},T^{-1}\right]\\ \left[\rho_{2}\right]=\left[M^{1},L^{0},T^{-2}\right] \end{array}$$

Then,

(4) $$\left|\begin{array}{ccc} 1 & -1 & -1\\ 0 & 1 & -1\\ 1 & -3 & 0 \end{array}\right|\neq 0$$

By solving the above equation, then *π*_1_ = *δ*/uη2, π1 = *gη*_2_/*uρ*_2_, could be obtained by the derivation.

(5) $$\frac{1}{\pi_{1}}=\frac{u\eta_{2}}{\delta }=Ca$$

The reciprocal of *π*_1_ is the ratio of the deforming stress exerted by the heavier phase and the counteracting Laplace pressure, and it is called the capillary number (Ca) [16,17,18,19,20,21,22].

At the same time:

(6) $$\frac{1}{\pi_{2}}=\frac{u^{3}\rho }{g\eta }=\frac{u^{2}\rho ul}{gl\eta }=Fr\cdot Re$$

The dimensionless Froude number (*Fr*) represents the ratio of momentum to buoyancy force. The Reynolds number (*Re*) is the ratio of inertia and viscous force.

(7) $$\pi_{3}=\rho_{1}/\rho_{2}$$

(8) $$\pi_{4}=\eta_{1}/\eta_{2}$$

As such, the general relation below can be expressed from the above equations:

(9) $$Ca=f\left(Fr\cdot Re,\frac{\rho_{1}}{\rho_{2}},\frac{\eta_{1}}{\eta_{2}}\right)$$

Finally, by observation, the capillary number can be written as the ratio of the Weber number to the Reynolds number, as shown below:

(10) $$Ca=\frac{u\eta }{\delta }=\frac{\rho u^{2}l}{\delta }\cdot \frac{\eta }{\rho ul}=We/Re$$

The Weber number represents the ratio of the momentum to interfacial tension force. Thus, the design of a water modelling experiment should make all the parameters (such as *We*, *Fr*, *Re*, *ρ*_1_/*ρ*_2_, *η*_1_/*η*_2_) similar simultaneously to properly simulate the steelmaking mold via water modelling.

For the high-velocity flow conditions present in a steel continuous caster, fully developed turbulent flow conditions are always produced, so achieving Reynold’s similarity by matching the ratio of the momentum and diffusion forces was judged to be less important, as long as fully turbulent flow conditions are maintained [2].

For the *Fr* and *We* similarity, the water model needs to satisfy the following equations:

(11) $$\frac{u_{steel}^{2}}{gL_{steel}}=\frac{u_{water}^{2}}{gL_{water}}$$

(12) $$\frac{u_{steel}^{2}L_{steel}\rho_{steel}}{\delta_{Fe-Slag}}=\frac{u_{water}^{2}L_{water}\rho_{water}}{\delta_{water-oil}}$$

where *L_steel_* is the actual size, *L_water_* is the characteristic length of water modelling; *ρ_steel_* is the molten steel density (7020 kg/m^3^), *ρ_water_* is the water density (998 kg/m^3^), and *δ_Fe-Slag_* is the surface tension, 1.2 N/m, for molten steel and slag [15], and *δ_water-oil_* depends on the oil used in the experiment. Applying a general scale factor of *λ* = *L_steel_/L_water_* in Equations (11) and (12), the combined scale factor should be:

(13) $$\lambda =\sqrt{\frac{\rho_{s}\cdot \delta_{w-o}}{\rho_{w}\cdot \delta_{Fe-Slag}}}$$

In this paper, a scale factor of 0.5 was determined according to the experimental conditions.

### 2.3. Experiment Schemes and Materials

Firstly, four cases, which were calculated using FLUENT, were as follows: A, submerged depth 70 mm, casting speed 1.6 m/min; B, submerged depth 70 mm, casting speed 1.8 m/min; C, submerged depth 90 mm, casting speed 1.6 m/min; D, submerged depth 90 mm, casting speed 1.8 m/min. Secondly, the best plan was calculated using the LES model with the slag layer. Finally, a water model was carried out and a high-speed camera was used to capture the oil droplet entrapments.

In this paper, water was used as the heavier phase. Silicone oil AK 0.65 was used as the lighter phase to develop a liquid–liquid interface with density 760 kg/m^3^ and interface tension 0.04 N/m, which behaves nearly like a Newtonian liquid.

### 2.4. Governing Equations and Boundary Conditions

**Figure 4:**
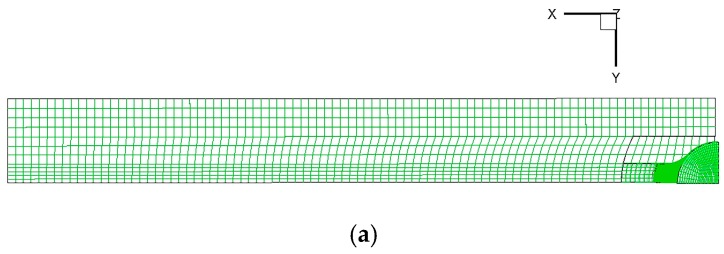

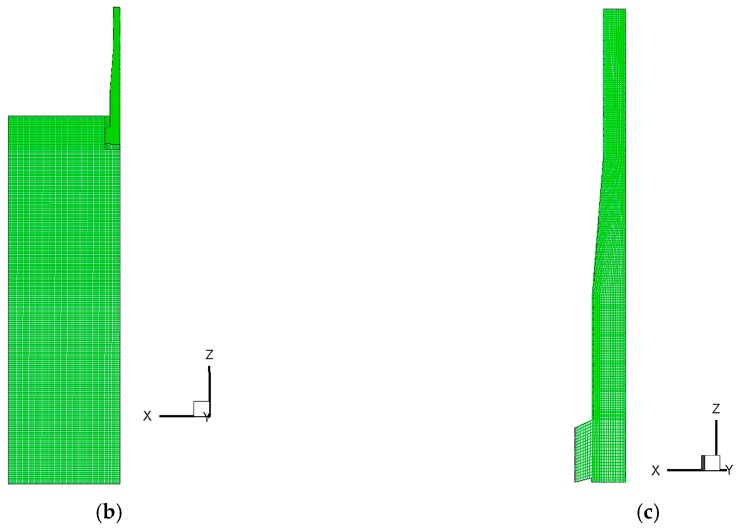
Schematic of meshes used for numerical simulation: (**a**) top view; (**b**) side view; (**c**) submerged entrance nozzle.

The commercial software FLUENT was used to solve the LES model using a DELL 8-core personal computer with 64.0 GB of random-access memory and a 3.00 GHz Intel® Xeon processor for parallel computing. The volume of fluid (VOF) model was used to track the interface between the phases. In this work, the tracking of the metal/slag interfaces was accomplished by solving a continuity equation for the volume fraction of the steel and slag phases. The LES model was also used to calculate the slag entrapment in the continuous casting mold. The fluid region in the entire mold of the continuous casting process was solved with 500,000 hexahedral structured meshes as shown in Figure 4.

The time of calculation was 15 s with a time step of 0.001 s. The finite volume method (FVM) was used to process the discrete data, and the SIMPLEC (Semi-Implicit Method for Pressure Linked Equations-Consistent) algorithm was used to solve the coupled equations of the FVM. The nozzle submergence depth was 90 mm, shown in Figure 3a. According to the casting speed of 1.6 m/min, the inlet velocity 1.329 m/s was back-calculated using continuity. The velocity inlet and pressure outlet were used. Steel/slag interface tension was 1.2 N/M [15].

## 3. Results and Discussions

### 3.1. Submerged Depth and Casting Speed

**Figure 5:**
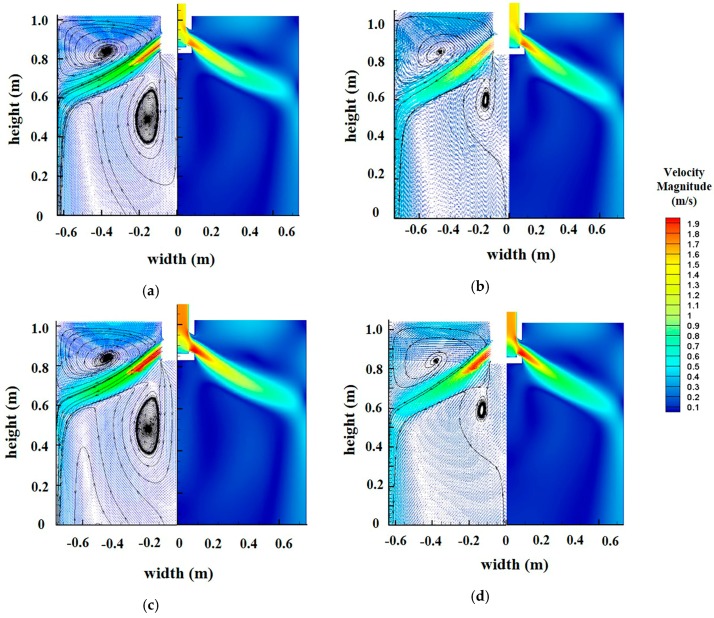
Contour and vector of velocity at wide face in mold: (**a**) casting speed 1.6 m/min, submerged depth 70 mm; (**b**) casting speed 1.8 m/min, submerged depth 70 mm; (**c**) casting speed 1.6 m/min, submerged depth 90 mm; (**d**) casting speed 1.8 m/min, submerged depth 90 mm.

The velocity magnitude field and vector of each scheme in the wide face of the mold was calculated via computational fluid dynamics (CFD) modelling, as shown in Figure 5.

**Figure 6:**
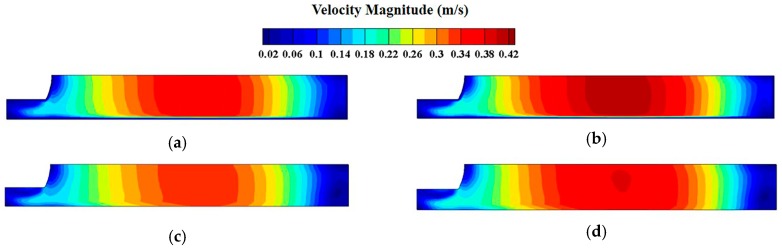
Contour of velocity magnitude at free-surface: (**a**) casting speed 1.6 m/min, submerged depth 70 mm; (**b**) casting speed 1.8 m/min, submerged depth 70 mm; (**c**) casting speed 1.6 m/min, submerged depth 90 mm; (**d**) casting speed 1.8 m/min, submerged depth 90 mm.

It could be seen that there are two strands of swirling flow in the mold during continuous casting: The upper part and the lower part. For the upper part, the influence of the velocity magnitude on slag entrainment is very important. When the flow rushes out of the nozzle port, it will move forward along the nozzle angle and then impact the narrow face, producing two streams. The upward flow will disturb the molten steel at the free surface, sometimes resulting in slag entrapment. The velocity magnitude field at the free surface is shown in Figure 6.

**Figure 7:**
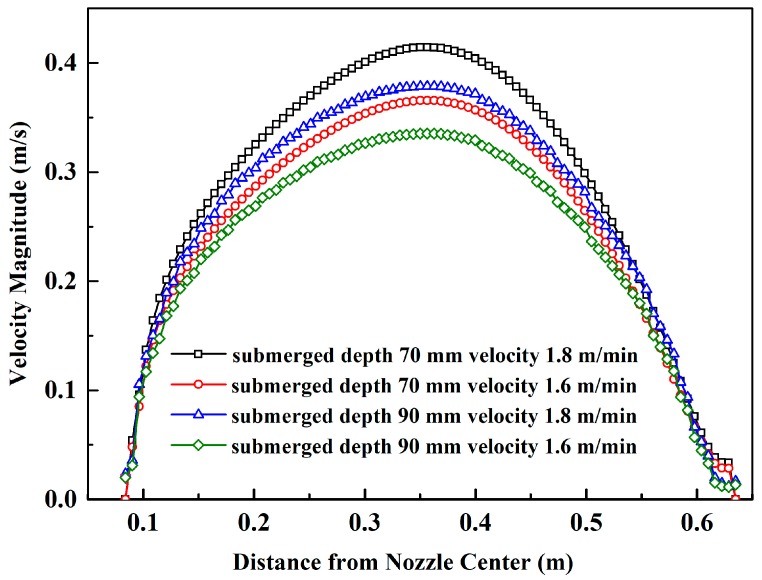
Velocity distribution of free-surface along the central line of the nozzle.

It could be seen that the velocity was highest at the mid-section of the free surface. The fluid with a high-speed velocity will impact the interface between molten steel and slag, so the higher the surface speed is, the easier slag entrapment occurs. To further compare the free-surface velocity of the four schemes, the velocity distribution along the central line of the nozzle center at free surface was plotted as Figure 7. Surface velocity reaches maximum when the location is 0.35 m away from the nozzle center of each scheme. And maximum surface velocity is smallest (0.335 m/s) when the submerged depth is 90 mm and casting speed is 1.6 m/min as shown in Table 1. Compared with the scheme of 70 mm, submerged depth and 1.8 m/min casting speed, the maximum surface velocity was reduced by 19.1% (from 0.414 m/s to 0.335 m/s). Even compared with the other two conditions in Table 1, the maximum surface velocity of 90 mm submerged depth and 1.6 m/min casting speed was still reduced by 11.4% and 8.3%, respectively.

Thus, the optimum scheme was determined: Submerged depth 90 mm and casting speed 1.6 m/min.

### 3.2. Slag Entrapment Ratio (SER)

**Figure 8:**
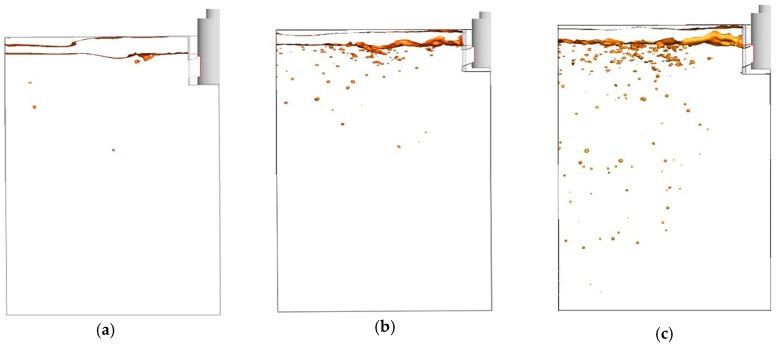
Slag droplets position at different times: (**a**) t = 5 s; (**b**) t = 10 s; (**c**) t = 15 s.

Based on the previous section’s discussion, we calculated the location of slag droplets at different times to study the behavior of the slag droplets involved in the conditions of submerged depth 90 mm and casting speed 1.6 m/min, as shown in Figure 8. From the results, we could find that the slag entrapment was clearly observed at t = 5 s due to the impact of the fluid on the steel slag interface. After that, new slag droplets were constantly involved in the molten steel as time went on. Slag droplets moved continuously toward the narrow face and a deeper molten steel area of the mold along with the movement of the stream. After 15 s, some of slag droplets already moved into deeper areas and some even moved to the wall position, which was likely to be captured by the solidified shell.

To explain the slag droplet entrapment in detail, we selected several cross sections along the longitudinal direction in the mold. Based on the simulation results, we defined the slag entrapment ratio (SER) in each cross section as follows:

(14) $$\varphi_{slag}=\frac{S_{slag}}{S_{slag}+S_{steel}}\times 100\%$$

**Figure 9:**
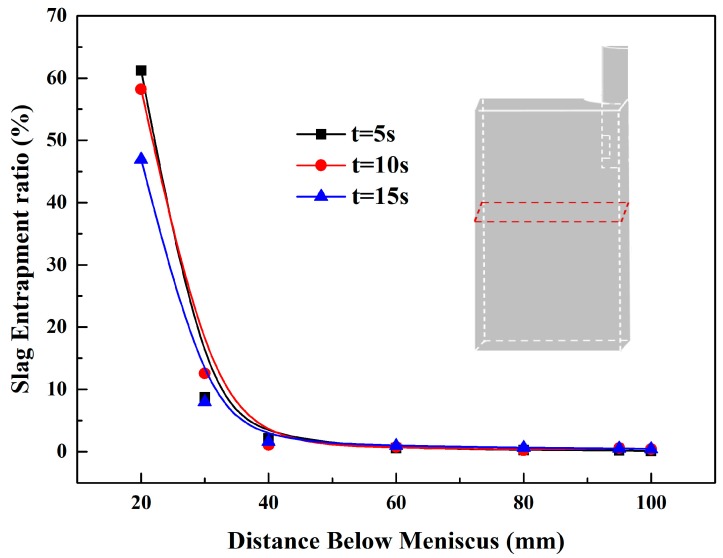
Slag entrapment ratio in each cross section.

Here, *S_slag_* stands for the slag phase volume fraction in each plane calculated by integral and *S_steel_* is the liquid steel phase volume fraction each plane calculated by integral. So, we can obtain the *φ_slag_* as Equation (14). Then, SER in the longitudinal was obtained, as shown in Figure 9.

It could be seen that, as time goes on, the area with a high slag entrainment ratio moves down gradually. The slag entrainment ratio below a meniscus of 0.02 m is the largest (61.23 % at t = 5 s), because it is the most drastic area of slag entrapment near the steel slag interface in mold. Then, the slag entrainment ratio begins to decrease after 15 s. This is caused by the slag droplets moving down with the swirling flow. At the same time, the phenomenon of slag entrapment reduces as the depth deepens. The slag droplets in the lower region were mainly derived from those from the upper region with the fluid flow to these areas. All the results reflected that location and the downward movement of the slag droplet. When slag droplets moved to the area 0.1 m below the meniscus, the largest SER is 0.44 % at t = 15 s. Thus, the value of SER is not high at any time which is consistent with reality.

### 3.3. Effect of Oil Used on the Water Modelling Experiments

Based on an analysis of Section 2.2, the density ratio of water to oil should be consistent with the density of molten steel to slag phase according to the Buckingham π theory. To satisfy the similarity of corresponding parameters in the theorem between water modelling and numerical simulation, the type of oil used in water modelling is crucial. To date, there is no clear standard to determine what kind of oil should be used in a water modelling experiment, so we studied the effects of oil used in the water model.

**Figure 10:**
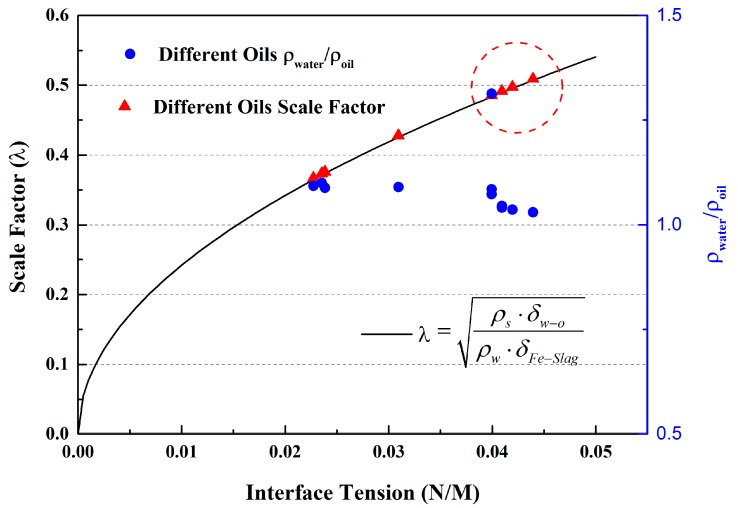
Relations between surface tension of different oil and scale factor.

Further to the density ratio, the surface tension of the oil used in water modelling is also an important factor for determining slag entrapment in the continuous casting process. Silicon oil and vegetable oil were studied by other researchers [23,24,25,26,27]. To find out the effects of oil on water modelling experiments, a relation curve was drawn between surface tension of different oils and scale factor as calculated by Equation (13), as shown in Figure 10.

By observation, it can be found that scale factor increases gradually with the increase of interfacial tension between oil and water. Thus, the scale factor corresponds according to the oil media chosen used in the water modelling experiment. In any case, we should take the density and viscosity of oil into consideration based on our results deduced from Buckingham *π* theory. For the continuous casting mold, the density ratio (*ρ_steel_*/*ρ_slag_*) was about 2.1~2.5. According to the parameters in Table 2, the density ratio *ρ_water_*/*ρ_oil_* of the liquid–liquid systems varied from 1.0 to 1.3, which is far more different than the value of 2.5 for steel–slag systems. No literature to date on water modelling of continuous casting molds uses any oil remotely near the ideal value of 2.5. Finding the optimum oil (satisfied with density ratio to water 2.5, while also being non-toxic and non-volatile) used in water modelling needs further investigation.

It could be seen from the table that oils in the red circle are the relatively good choices, because the scale factor is about 0.5 and that is convenient for making a water model according to the Equation (13). At the same time, the density ratio of the oil used (silicon oil AK 0.65) in the experiment was the closest one compared with other oils in the Table 2. Thus, it is a better choice to use silicon oil AK 0.65 to simulate the slag phase in the water modelling experiment. This could be used as a reference for how to determine λ and oil used in water modelling experiment for slag entrapment in molds.

### 3.4. Model Validation

**Figure 11:**
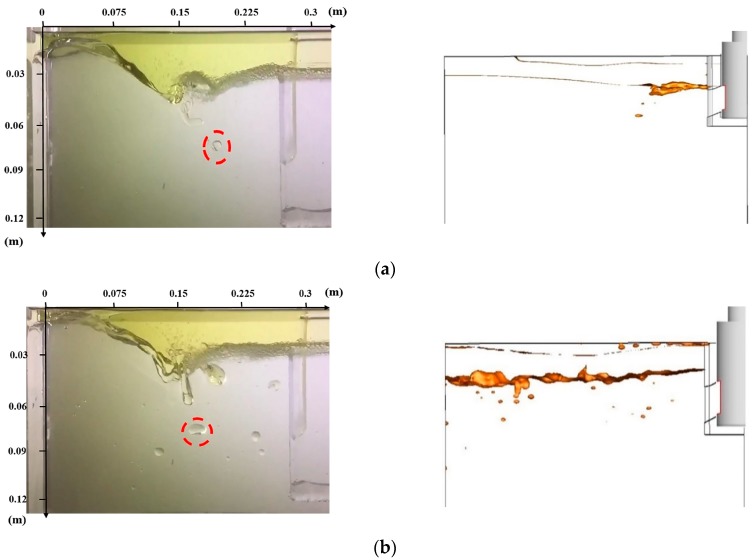
Comparison of water simulation experiments and numerical results: (**a**) t = 5 s; (**b**) t = 10 s.

We compared the results of the water modelling with the numerical simulation results, as shown in Figure 11.

We could find that the water modelling results are in good agreement with the numerical simulation results. The coincidence between the water modelling results and the simulation results is high, no matter the position of slag entrainment or the shape and size of slag droplets being involved into molten steel. Therefore, the results of numerical simulation can provide us with an effective way of better understanding the occurrence of slag entrapment in a continuous casting process.

**Figure 12:**
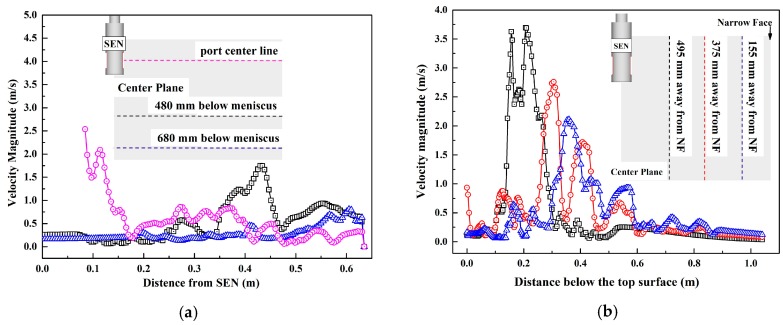
Velocity distribution of different lines at several locations: (**a**) horizontal variations; (**b**) vertical variations.

To further understand where slag droplets are likely to appear in molds, we draw the velocity curves of different lines at several locations, as shown in Figure 12. From Figure 12a, velocity can be seen to decrease with increasing distance from the SEN port. Along the center line of the nozzle port, the velocity decreased first then began to fluctuate due to the upper circle flow and turbulence. The velocity curve increases with the distance away from the nozzle. Therefore, the slag droplets in the red circle marked in Figure 11a,b both are near the area of the cross section of port center line and away from the SEN (0.1 m). As the velocity peak appears (2.0 m/s), slag droplets are more likely to be entrapped in this area.

From Figure 12b, it could be seen that the farther the distance is, the farther the velocity peak appears. At the same time, the maximum velocity decreases with the increase of distance. Thus, these velocity distributions could tell us which areas we should focus on.

## 4. Conclusions

In this paper, the optimum submerged depth and casting speed in a 150 × 1270 mm slab continuous casting process were studied by numerical simulation. Then, the slag entrapment behavior in a continuous casting mold was validated by using numerical calculation and water modelling methods. The following conclusions can be drawn from this work:

1. The optimum scheme is SEN submerged depth is 90 mm and casting speed 1.6 m/min. The maximum surface velocity of the optimum scheme is the smallest (0.335 m/s), which is reduced by 19.1%, 11.4% and 8.3%, respectively, compared with the other three conditions.
2. A new dimensionless parameter, the slag entrapment ratio (SER), was defined to explain the mixing of slag phase in each cross-plane. After 15 s, the maximum SER (0.44 %) appeared at the position of 0.1 m below the meniscus.
3. The dimensionless parameter density ratio was found to be an important factor. Furthermore, the density ratio of oil used (silicon oil AK 0.65) in the water modelling experiment to water is closer to the density ratio of slag and steel compared with other common oils.
4. The water modelling results were in good agreement with the numerical simulation results and the slag droplets observed in the experiment were found to be more likely to be entrapped in the area where the SER value is large.

## Figures and Tables

**Table 1:** Maximum surface velocity of different submerged depth and casting speed (m/s).

70 mm 1.8 m/min	90 mm 1.8 m/min	70 mm 1.6 m/min	90 mm 1.6 m/min
0.414	0.379	0.366	0.335

**Table 2:** Properties of different oils [15,27].

Oil	Density (kg/m^3^)	Interface Tension (N/m)	Density Ratio (*ρ_water_*/*ρ_oil_*)
Silicon oil AK 0.65	760	0.04	1.313
Silicon oil AK5	920	0.04	1.085
Silicon oil AK10	930	0.04	1.073
Silicon oil AK35	955	0.041	1.045
Silicon oil AK50	960	0.041	1.040
Silicon oil AK100	963	0.042	1.036
Silicon oil AK200	966	0.044	1.033
Silicon oil AK500	969	0.044	1.030
Peanut oil	916	0.031	1.090
